# Nondestructive X-ray diffraction measurement of warpage in silicon dies embedded in integrated circuit packages^1^

**DOI:** 10.1107/S1600576717003132

**Published:** 2017-03-22

**Authors:** B. K. Tanner, A. N. Danilewsky, R. K. Vijayaraghavan, A. Cowley, P. J. McNally

**Affiliations:** a Department of Physics, Durham University, South Road, Durham, County Durham DH1 3LE, UK; b Kristallographie, Albert-Ludwigs-Universität, Freiburg, Germany; c School of Electronic Engineering, Dublin City University, Dublin 9, Ireland

**Keywords:** X-ray diffraction imaging, encapsulated silicon dies, warpage

## Abstract

Transmission X-ray diffraction imaging in both monochromatic and white beam section mode has been used to measure quantitatively the displacement and warpage stress in encapsulated silicon devices. The displacement dependence with position on the die was found to agree well with that predicted from a simple model of warpage stress. For uQFN microcontrollers, glued only at the corners, the measured misorientation contours are consistent with those predicted using finite element analysis. The absolute displacement, measured along a line through the die centre, was comparable to that reported independently by high-resolution X-ray diffraction and optical interferometry of similar samples. It is demonstrated that the precision is greater than the spread of values found in randomly selected batches of commercial devices, making the techniques viable for industrial inspection purposes.

## Abstract 2

X-ray diffraction imaging in both monochromatic and white beam section mode has been used to measure quantitatively the displacement and warpage stress in encapsulated silicon devices.

## 1. Introduction   

Future integrated chip manufacturing must encompass complex chip systems capable of diverse functionality and application, so-called ‘more than Moore’ manufacturing. Such systems include microelectromechanical systems, system in package, system on chip and three-dimensional integrated circuits, referred to collectively as ‘advanced packages’. This technology requires processing of thin semiconductor dies (25–100 µm thickness) and many packages include multiply stacked silicon dies. Because the wafers are thin, they may be subjected to substantial elastic strain and displacement without fracturing. Thus, during both processing and encapsulation there is a serious risk of failure through macro-scale short circuiting. The development of local stresses in the die can also lead to altered performance of surrounding transistors (*e.g.* stress-induced carrier mobility variations) and stress-sensitive structures. Current practice avoids these by the placement of on-die ‘keep out zones’. These represent wasted ‘real estate’ and their reduction or elimination is desirable. Further, packages subjected to high temperature may fail owing to flow of polymer and the associated wafer movement. A recent review (Marks *et al.*, 2014 ▸) revealed that none of today’s commercially available metrology tools is capable of measuring, *in situ* and non-destructively across an entire die, the nature and scale of wafer/die strain or bow/warpage. Many techniques are destructive and those that are nondestructive tend to measure the package bow which, crucially, is not the same as the wafer/die bow.

Toda & Ikarashi (2010 ▸) showed that the warpage of LSI (large-scale integration) chips, mounted on a printed circuit and moulded with resin, could be measured by high-resolution X-ray diffraction in reflection geometry using 17.5 keV (Mo *K*α) radiation. From the displacement of the Bragg peak position across the wafer, the strain and macroscopic warpage were deduced. Similar measurements, in reflection geometry, have been reported by Wong *et al.* (2014 ▸) on thin-package commercial QFN (quad flat non-lead) chips using Cu *K*α radiation. More recently, the distortion of white radiation transmission section topographs has been used with B-spline fitting techniques to map the warpage in commercial packaged chips (Cowley *et al.*, 2016 ▸). With synchrotron radiation, these measurements are extremely rapid (Bose *et al.*, 2016 ▸).

Since Renninger’s pioneering experiments in the 1960s (Renninger, 1962 ▸), monochromatic plane wave imaging has been used to create maps of effective misorientation of distorted wafers. From such a specimen, only a narrow stripe of intensity is recorded, corresponding to the spatial limit of the sample oriented within the width of the rocking curve. Renninger (1962 ▸, 1965*a* ▸,*b* ▸) showed that, by angularly displacing the sample with respect to the incident beam direction and superimposing successive images, what he called a ‘zebra pattern’ resulted, mapping contours of effective misorientation. By reversal of the diffraction vector or exchange of input and exit surfaces, tilt and dilation can be separated, and the technique has been applied to Si (Jacobs & Hart, 1977 ▸; Hart, 1981 ▸; Ishikawa *et al.*, 1985 ▸) and GaAs (Barnett *et al.*, 1987 ▸). With current digital imaging systems the construction of the ‘zebra patterns’ is now extremely straightforward. We show in this paper that the technique can be used to measure the warpage in packaged chips and compare the method with the section topography technique. While the local effective misorientation can be measured accurately at typically micrometre spatial resolution by analysis of individual pixels in the image (Lübbert *et al.*, 2000 ▸), such precision, with the associated penalty in data collection time, is not necessary for the macroscopic curvature studied here.

## 2. Experimental technique   

Diffraction imaging was performed on beamline B16 at the Diamond Light Source in the UK. All images were recorded using the Photonic Science X-ray MiniFDI, camera which has an 11 mm diameter input window and 1392 × 1040 pixels (6.5 µm optical pixel size). Increasing the Bragg angle corresponds to an upward vertical shift of the image, and in all figures presented herein, the projection of the diffraction vector is vertically up the page. *ImageJ* (https://imagej.nih.gov/ij/) was used for image processing. The white X-ray beam was collimated into a very thin pencil-shaped beam, whose vertical height was typically 15 µm and whose length was up to the 10 mm limit on the beamline aperture. Such a narrow beam is essential in order to eliminate any potential heating effects of the X-ray beam on the chip package. (Previous experiments using the full available beam area resulted in irreversible damage to the samples.) For usable intensity, the maximum energy in the principal reflection in the section topographs was 46 keV, this being the limit of the spectral output of the bending magnet on B16. For the monochromatic experiments, the water-cooled, independently tuned, Si(111) double-crystal monochromator provided a beam of area typically 4 × 7 mm and of maximum energy 24.25 keV, close to the wavelength of the Ag *K*β line. [Although a water-cooled Si(311) channel-cut monochromator is available at B16 which will deliver a beam of energy out to about 35 keV, the effect of the residual heat bump was to reduce the intensity to a level at which the signal to noise ratio for the diffraction stripe was unacceptable.] Symmetric 220 reflections in transmission geometry were the reflections selected from the encapsulated (001) die.

## 3. Results   

### 3.1. Monochromatic beam   

**Figure 1:**
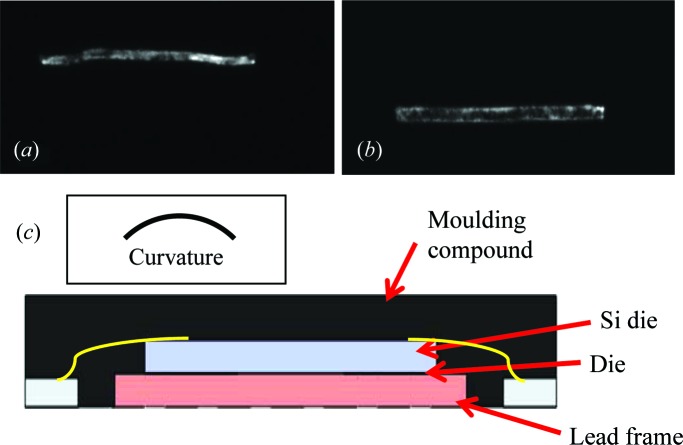
(*a*), (*b*) Diffracted images from the uQFN encapsulated chip on displacement of the sample by 0.15° across the rocking curve. Contact pads on entrance surface. 220 reflection. (*c*) Schematic diagram of integrated circuit package and deduced curvature of the silicon wafer.

Figs. 1 ▸(*a*) and 1 ▸(*b*) show the diffracted beam stripe from the wide-area monochromatic beam recorded from diffraction through a 28-pin uQFN flash microcontroller (manufacturer part No. PIC16LF1827-I/MV) from Microchip. The fully encapsulated package, of overall dimension 10 mm × 10 mm × 160 µm, contains a 5 × 5 mm active die bonded (001) Si chip (50 µm thick), embedded face-up on a substrate. There is a positive 0.15° rotation of the sample between Figs. 1 ▸(*a*) and 1 ▸(*b*). As the diffracted region moves down the sample, we can deduce that the curvature is concave when viewed from the surface on which the contact pads have been deposited (Fig. 1 ▸ *c*). Reversal of the exit and entrance surfaces results in the stripe moving upwards on an increase of the sample angle across the rocking curve.

**Figure 2:**
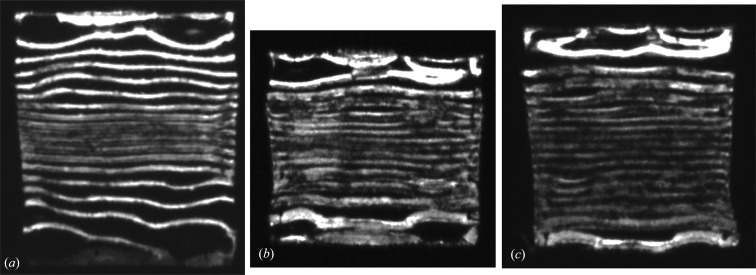
(*a*) Zebra pattern formed by summing images across the rocking curve displaced by 0.03°. 220 reflection. (*b*) Second sample which is rotated (*c*) by 90° about the surface normal.

**Figure 3:**
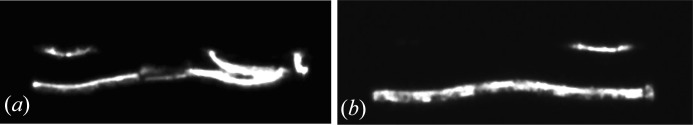
Displacement of sample by 0.03° between (*a*) and (*b*) results in reverse movement of the subsidiary contour around the die corner edge compared with the main wafer curvature.

The adhesive used to attach the chip to the lead frame is applied only at the four corners of the square wafer and cured at elevated (>373 K) temperature. The thermal contraction on cooling to room temperature placed a biaxial compressive strain on the silicon wafer, as the thermal expansion of the lead frame is an order of magnitude greater than that of silicon (Wong *et al.*, 2014 ▸). The superimposed ‘zebra pattern’ images of Fig. 2 ▸ show that there is a uniform curvature across the main body of the die of 0.51 ± 0.03°, that is 0.25° on either side of the centre. Rotation of the sample by 90°, about the [001] direction normal to the die surface, results in a similar map of the contours of effective misorientation (Fig. 2 ▸ *c*). The total data collection time for the 20 images of the full zebra pattern map in Fig. 2 ▸ was 75 s at B16 of the Diamond Light Source. Finite element simulations (Wong *et al.*, 2014 ▸) show a uniform curvature in the two orthogonal directions, with a negative gradient in the effective misorientation when approaching the four points where the wafer is glued to the lead frame. This is what is seen in the experimental misorientation contours. Although it is not clear in the full zebra pattern, inspection of individual images (Fig. 3 ▸) shows that the contours in the vicinity of the corner regions are displaced in the opposite direction to the main contours on rotation of the sample.

### 3.2. White beam section topography   

**Figure 4:**
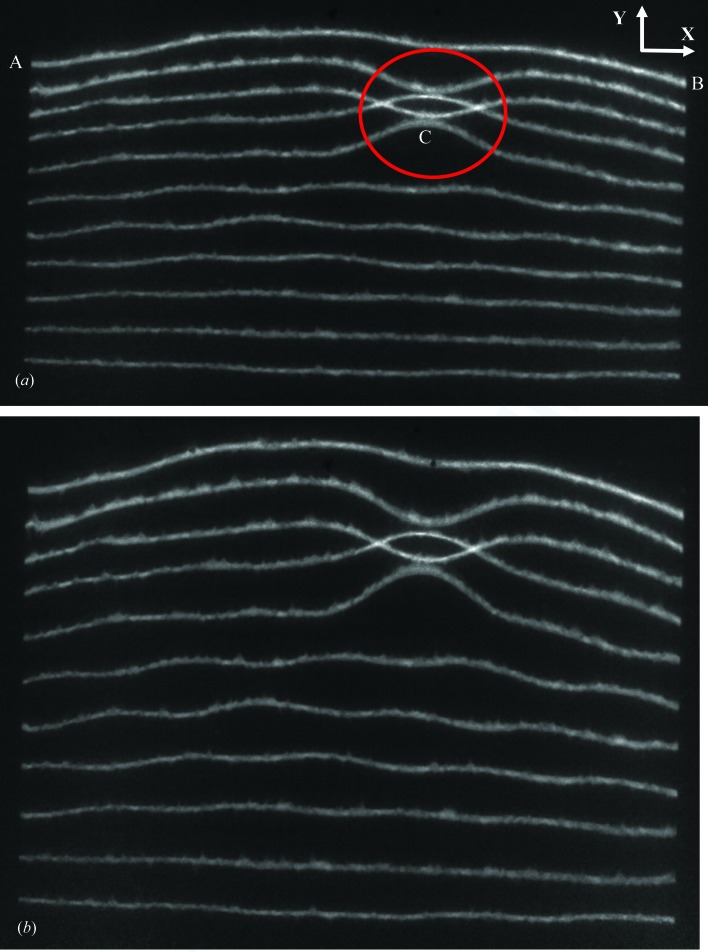
Section images from different regions of a 10 mm × 10 mm × 180 µm die glued to an FR4 frame but not encapsulated with epoxy. The successive lines are the diffraction images obtained as the die is displaced successively by 0.5 mm in the **Y** direction. Specimen to detector distance (*a*) 177 mm, (*b*) 277 mm. 220 reflection, Bragg angle 4°, principal wavelength 0.027 nm. The projection of the diffraction vector onto the image is, as in all cases, vertically up the page.

The section topography (imaging) technique was originally invented by Lang (1958 ▸) in order to probe, with a ribbon beam typically 15 µm wide, the lattice perfection of a virtual section through a crystal. While the low Bragg angles associated with high-energy diffraction make this original use only applicable to very thick crystals, for thin warped crystals the method provides a rapid way of quantitatively measuring the warpage. Because the white synchrotron radiation beam from a bending magnet has very low divergence but high polychromaticity, depending on the orientation of any point on the sample, a specific wavelength will be selected for diffraction. The result for very thin packaged dies is a very large distortion of the nominally straight line image of the 15 µm slit before the sample (Cowley *et al.*, 2016 ▸; Bose *et al.*, 2016 ▸). In the example shown in Fig. 4 ▸, the centre of the encapsulated wafer is undistorted, while there is significant warpage at the upper edge. These images are a superposition of a series of section images taken as the wafer is displaced laterally in 0.5 mm steps in the **Y** direction. Data collection at the Diamond Light Source is rapid, individual images being recorded in about 500 ms, and enough data to reconstruct a complete warpage map of an entire 2.2 mm × 2.4 mm × 150 µm Si die can be acquired in 50 s.

The horizontal straight-line image at the bottom of Fig. 4 ▸(*a*) indicates almost zero warpage, and the initial steps across the wafer remain parallel and of equal displacement. By the time the wafer has been displaced 2.5 mm from its centre, both short-range and long-range lattice displacement is evident across the wafer. There are striking local variations such as that circled in red. At the top of the wafer, 5 mm from its centre, there is an image displacement of 0.56 ± 0.01 mm (in the **Y** direction) between the edge and centre of the wafer (in the **X** direction). With a specimen to detector separation of 177 mm, this linear displacement at the detector corresponds to a tilt angle difference between the wafer edge and centre (in the **X** direction) of 0.1° in the diffraction plane, *i.e.* measured in the **Y** direction. (Because it is the result of a wavelength change, the angular displacement of the diffracted beam is twice that of the sample.) The precision on this measure is poor, however, because the absolute distance between detector and specimen is difficult to determine.

**Figure 5:**
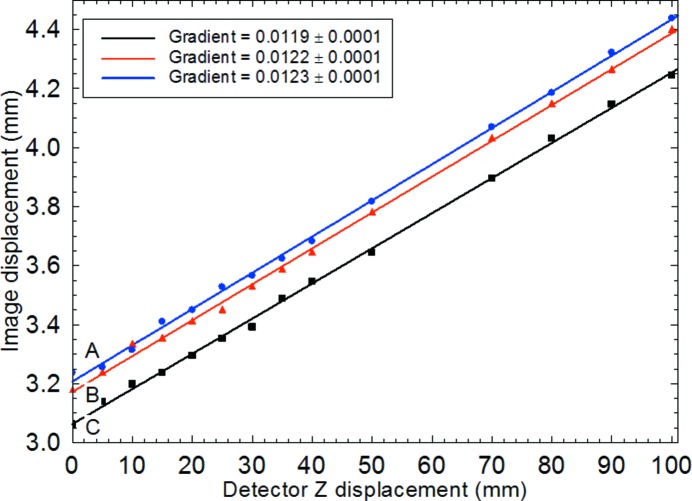
Image position of features A, B and C in Fig. 4 ▸ as a function of sample–detector distance. 4° Bragg angle, 0.027 nm principal wavelength.

The tilt can be determined precisely by measurement of the position of successive section images on displacement of the detector, with respect to the sample, in the **Z** direction, parallel to the incident X-ray beam. An example of the corresponding change in image position of points A, B and C from Fig. 4 ▸ is given in Fig. 5 ▸. Here, the distance of each point with respect to the section image from the undistorted centre of the wafer, *i.e.* the bottom image in Fig. 4 ▸, is plotted as a function of relative detector displacement. This provides a measure of the tilt measured in the **Y** direction at each point with respect to the wafer centre. The tilt at point A of Fig. 4 ▸ is 0.352 ± 0.003°, at point B it is 0.350 ± 0.003° and it is 0.341 ± 0.003° at point C.

**Figure 6:**
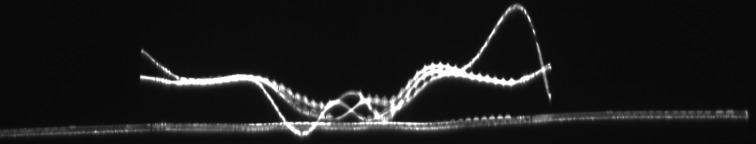
White beam section topographs of a package containing four stacked Si dies. The three top dies are 5 mm × 5 mm × 50 µm thick, while the bottom die is 8 mm × 8 mm × 200 µm thick. 220 reflection.

Toda & Ikarashi (2010 ▸) showed that, using high-energy high-resolution X-ray diffraction, the wafer warpage could be determined from the rocking curve position not just for one die but also for multiply stacked dies. They gave an example of the three rocking curves that could be observed from three stacked interconnected dies and how the wafer bow could be tracked independently for each wafer from the single measurement. Similarly, multiple die stacks yield multiple section images. Fig. 6 ▸ shows an example of an un-encapsulated interconnected four-die stack in which the three top dies are 5 mm × 5 mm × 50 µm thick, while the bottom die is 8 mm × 8 mm × 200 µm thick. The thick bottom die is almost undistorted, while the top three thin dies are very strongly warped by the interconnect and die adhesion processes.

## 4. Discussion   

**Figure 7:**
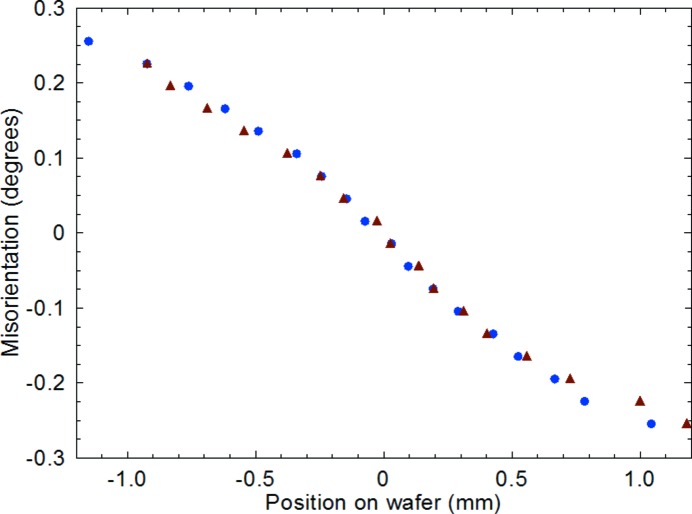
Effective misorientation as a function of position across the uQFN die in a direction parallel to the diffraction vector. Triangles and squares correspond to measurements on the same die but with the sample rotated 90° about the surface normal.

**Figure 8:**
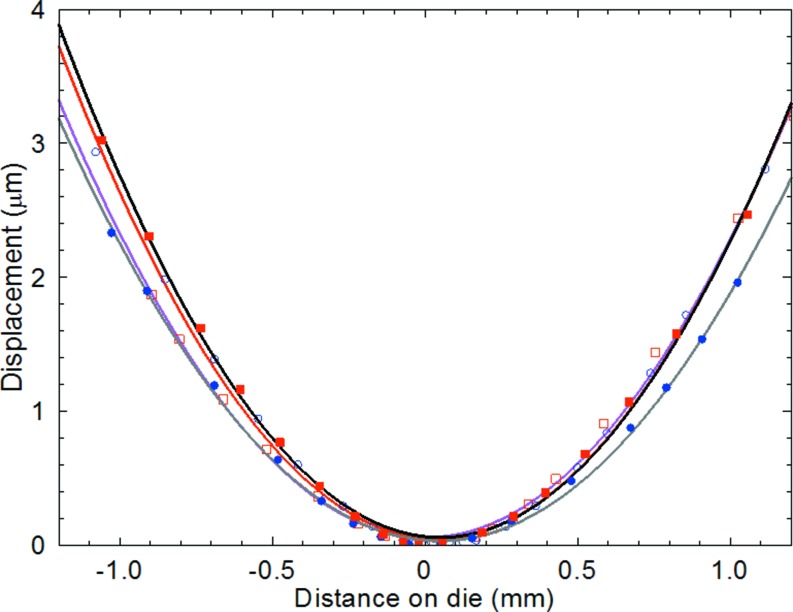
Displacement of two uQFN packaged dies (open and closed data points) as a function of distance from the die centre. Squares and circles correspond to measurements on the same die but with the sample rotated 90° about the surface normal. Solid lines are quadratic fits to the data.

In all the packages examined, the die is curved in a convex shape with respect to the lead frame. This arises from thermal contraction of the lead frame on cooling to room temperature after application and curing of the adhesive at elevated temperature. We have already noted that the features at the edges of the contour maps of the uQFN microcontroller die (Fig. 2 ▸) are in qualitative agreement with those predicted in the finite element modelling reported by Wong *et al.* (2014 ▸). In addition, from Fig. 2 ▸, we can quantify the magnitude of the main convex bending of the wafer giving rise to the dominant movement of the diffraction stripes on sample rotation. Fig. 7 ▸ shows the effective misorientation as a function of position for a die chosen at random from a commercial batch. The two curves correspond to the 220 and 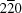 reflections, *i.e.* they measure the curvature in the two orthogonal directions in the die surface. The variation is almost linear, the deviation being related to the two-dimensional nature of the compression induced on cooling to room temperature following fixing and curing of the adhesive at the four corners. Integration of the misorientation with distance from the wafer centre gives the warpage (Fig. 8 ▸), that is, the displacement of the point from the undistorted plane of the die. This is almost parabolic, in qualitative agreement with the warpage reported previously by Toda & Ikarashi (2010 ▸). The solid lines in Fig. 8 ▸ are parabolic fits to the displacement in a line parallel to the **Y** (wafer edge) direction through the wafer centre.

The stress in a beam bent into a convex shape is tensile in the upper surface and compressive in the lower surface, there being a line of zero stress at the mid-point. The warpage stress σ can readily be shown to be related to the die thickness *t* and the difference in lattice plane orientation Δω_B_ between points separated by distance Δ*d* across the die surface. *E* is Young’s modulus of silicon. The result is (Toda & Ikarashi, 2010 ▸)
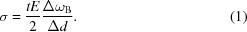
While Young’s modulus is not a simple scalar quantity owing to the elastic anisotropy, the value for bending in the 〈110〉 direction of a (001) die has been shown to be *E* = 170 GPa by Hopcroft *et al.* (2010 ▸). As the uQFN die is 2.2 × 2.4 mm by 150 µm thick, we have from Figs. 7 ▸ and 8 ▸ values of 58.7, 54.8, 57.4 and 48.5 (± 2) MPa for the warpage stress σ from the four independent measurements. The linear variation of misorientation with distance results in a constant value of σ, consistent with the finite element modelling results of Wong *et al.* (2014 ▸), who predicted a general constant warpage stress in both of the orthogonal directions. The values reported here are of the same magnitude but somewhat smaller than those predicted and measured by Wong *et al.* (2014 ▸) using high-resolution X-ray diffraction in the reflection geometry. It is evident, however, that the spread of values reported above for different samples orientation is greater than the measurement precision, indicating that the method has potential for quality control applications.

The die used to record the section images of Fig. 4 ▸ was of dimension 10 mm ×10 mm × 180 µm and was from IMEC, glued to an FR4 (glass-reinforced ep­oxy laminate) substrate at 388 K and subsequently cooled to room temperature. A manually dispensed no-flow under-fill, with thickness between 0.07 and 0.1 mm, was used to secure the die on the substrate. This sample was not encapsulated in epoxy resin, making optical profilometry possible.

**Figure 9:**
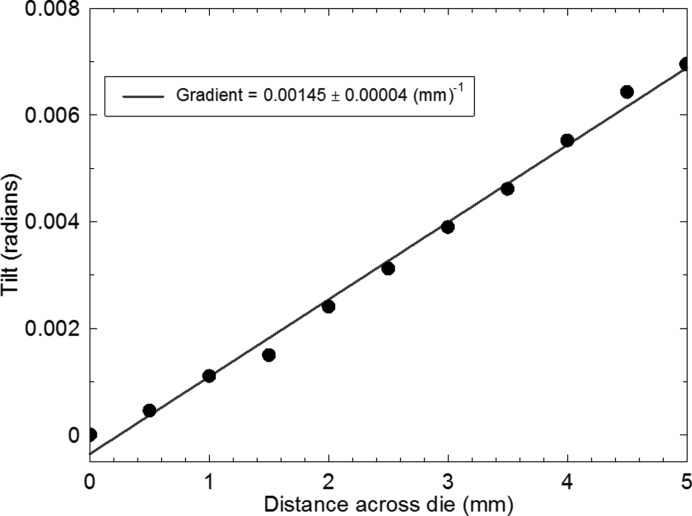
Tilt of points along a line through the centre of the die as a function of position in the **Y** direction. The solid curve is a linear fit to the data.

**Figure 10:**
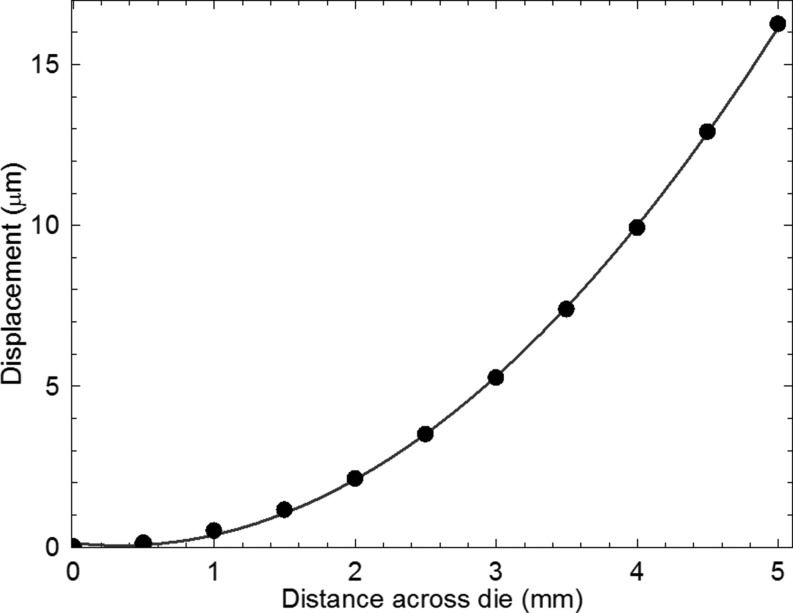
Displacement of points in a line through the centre of the die as a function of position along the die obtained by integration of tilt measurements. The solid line is a quadratic fit to the data.

Having established that the image displacement is linear with specimen to detector distance (Fig. 5 ▸), we can determine the tilt as a function of position along the die by subtraction of the image distance from the central baseline for just two specimen to detector distances, for example as in Fig. 4 ▸. As a function of position across the die, along a line parallel to the **Y** direction, we find a linear variation (Fig. 9 ▸) both through the die centre and along the die edge. The trend is more nearly linear than for the uQFN package measured in the monochromatic beam experiments. Integration of these curves gives a parabolic variation of warpage displacement as a function of position (Fig. 10 ▸). The absolute displacement, measured along a line through the die centre, was 16 ± 0.1 µm at the die extremity, comparable to that of 11 µm reported by Toda & Ikarashi (2010 ▸) for a 9 mm packaged die. It is also consistent with the parabolic profile and maximum warpage displacement of 13.5 ± 0.6 µm measured by optical interferometry on a similar sample (Bose *et al.*, 2016 ▸). Using equation (1), the warpage stress σ was determined to be 22.2 ± 0.6 MPa from the gradient of Fig. 9 ▸.

**Figure 11:**
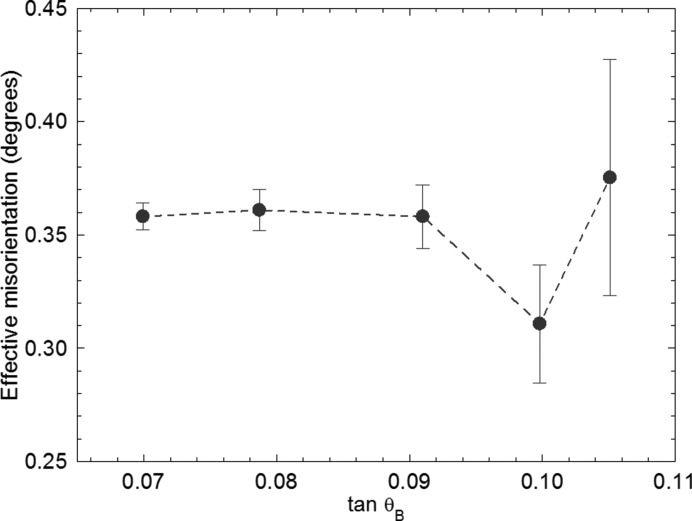
Effective misorientation at point A in Fig. 4 ▸, measured at different wavelengths. There is no systematic trend with Bragg angle.

A major challenge to X-ray techniques for measuring die warpage is that, unless an analyser is used in the optical configuration, the change in Bragg angle determines the effective misorientation, which is a combination of tilt and dilation. As the X-ray wave in the Bragg (reflection) geometry does not penetrate the whole crystal thickness, high-resolution diffraction measurements in this geometry will sense the warpage strain at the convex surface of the Si die. As the penetration is dependent on the curvature and the strain varies with depth, the measurement precision falls. The effect is mitigated by use of high-energy X-rays, such as described by Toda & Ikarashi (2010 ▸) where the small Bragg angle reduces the sensitivity to strain, but the complication is not completely removed. On the other hand, use of transmission experiments results in sensitivity only to the warpage tilt. Because the strain through the die thickness is tensile in the upper half of the die and compressive in the lower half, the strain averages to zero and only the tilt of the lattice planes contributes to the change in Bragg angle. The absence of strain sensitivity can be verified by measuring the tilt at a particular point as a function of wavelength. An example is shown in Fig. 11 ▸, and indeed there is no systematic variation with wavelength.

**Figure 12:**
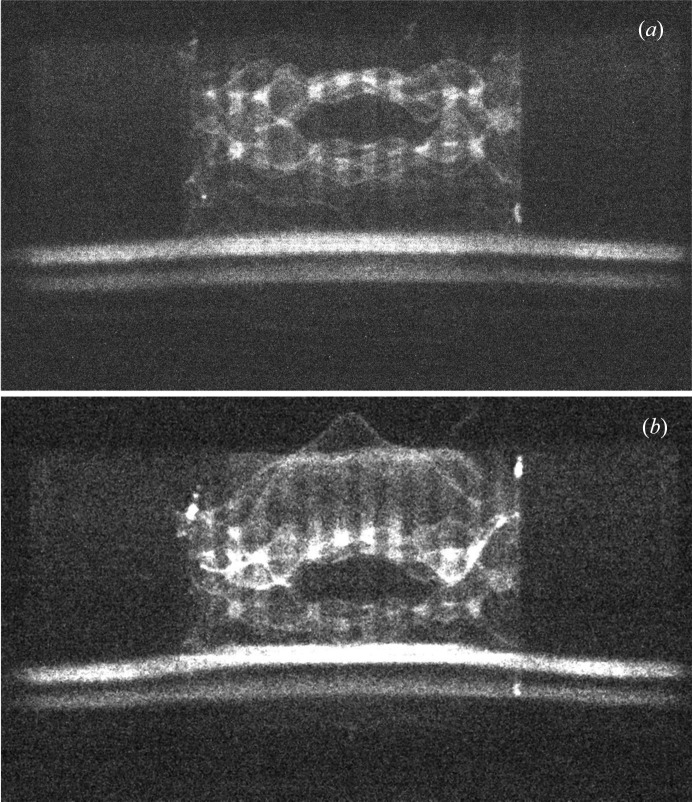
Monochromatic section topographs of the four-wafer un-encapsulated stack imaged in Fig. 6 ▸. Image width 8 mm. 220 reflection, Mo *K*α_1_ and Mo *K*α_2_ wavelengths taken on a Bruker X-ray UK JVQC-TT diffraction imaging tool. Image (*b*) is with the sample displaced by 1 mm from the position for image (*a*).

While the intensity available at synchrotron radiation sources makes either technique very attractive in terms of throughput and a viable inspection tool, if the method is to become widely accepted, it is essential that either an in-fab (*i.e.* within the manufacturing facility) or an in-line tool be developed. At a synchrotron radiation source, the beam is highly parallel but contains a wide range of X-ray energies. From a conventional X-ray tube one has a large angular divergence but narrow characteristic lines in the spectrum. Thus, in principle, it is possible to make equivalent measurements from the distortion of a section topograph from a conventional source. X-ray diffraction imaging tools are on the market, for example, the Bruker X-ray UK JVQC-TT instrument. This is based on the BedeScan concept originally developed by Bowen *et al.* (2003 ▸) and subsequently successfully patented (Bowen *et al.*, 2004 ▸). We have undertaken successful preliminary trials on one of these instruments, which conventionally uses Mo *K*α radiation. With this wavelength, the absorption was too large to obtain acceptable quality data from the fully encapsulated wafers that we studied. However, images from the package of four un-encapsulated stacked dies shown in Fig. 6 ▸ had a better signal to noise ratio. Fig. 12 ▸ shows two section topographs of this sample, taken on a Bruker Semi UK JVQC-TT diffraction imaging tool, displaced by 1 mm across the wafer. For the images of Fig. 12 ▸, the Bruker tool was configured such that the slit close to the sample was 3 mm in width, with the slit in front of the X-ray tube 0.15 mm in width. As the X-ray source itself is 1.2 mm wide, the divergence was determined by the slit widths and their separation of 255 mm. The resulting 0.7° divergence was sufficient to cover the diffraction from the 8 mm long, 200 µm thick wafer at the bottom of the image and the distorted images from the three 5 mm long, 50 µm thick wafers at the top. (The diffuse vertical stripes are associated with absorption in the woven FR4 lead frame.)

The Mo *K*α_1_ and Mo *K*α_2_ images of the 200 µm wafer are, as expected from Fig. 6 ▸, relatively undistorted, while those of the 50 µm wafers are complex and not identical. Operating the tool in high-resolution static mode gives images from the two wavelengths corresponding to different positions on the sample. Interpretation is further complicated by caustics from the *Bremsstrahlung* and therefore is not straightforward for highly distorted wafers such as shown in Figs. 6 ▸ and 12 ▸, and further work is needed to devise a data reduction strategy. Simple replacement of the Mo X-ray source in the Bruker X-ray UK JVQC-TT instrument by one, commercially available, with an Ag target will give 21.99 (*K*α_2_) and 22.16 keV (*K*α_1_) characteristic lines which are of comparable energy to the radiation used at the Diamond Light Source. This should enable transmission measurements of fully encapsulated wafers to be undertaken rapidly in a production environment with data quality comparable to that of Fig. 12 ▸.

## 5. Conclusions   

Both plane wave monochromatic and white beam section imaging can be used in transmission mode to determine quantitatively the warpage in Si dies in encapsulated microelectronic devices. Use of the transmission geometry results in the effective misorientation being sensitive only to tilt, thereby enabling the macroscopic die warpage to be determined directly from either the zebra pattern contours or the dis­place­ment of the section topography images. The precision is such that it exceeds the sample to sample variation in randomly selected commercial sample batches. At the synchrotron radiation source, the data collection time is very attractive for rapid statistical inspection of devices, but for wide adoption, the techniques must be translated into the silicon fabrication (fab) environment. While a conventional X-ray tube is unlikely to deliver sufficient intensity into a broad monochromated high-energy X-ray beam for the monochromatic technique to be viable as a quality control tool in an industrial environment, the transmission section imaging method does look promising.
